# LTR-Net: A deep learning-based approach for financial data prediction and risk evaluation in enterprises

**DOI:** 10.1371/journal.pone.0328013

**Published:** 2025-08-01

**Authors:** Shimiao Liu

**Affiliations:** Changchun University of Finance and Economics, Changchun, Jilin, China; University of Wisconsin-Eau Claire, UNITED STATES OF AMERICA

## Abstract

Financial data prediction and risk assessment represent a complex multi-task problem that requires effective handling of time-series data and multi-dimensional features. Traditional models struggle to simultaneously capture temporal dependencies, global information, and intricate nonlinear relationships, resulting in limited prediction accuracy. To address this challenge, we propose LTR-Net, a multi-module deep learning model that combines LSTM, Transformer, and ResNet. LTR-Net effectively processes the multi-dimensional features and dynamic changes in financial data by incorporating a temporal dependency modeling module, a global information capture module, and a deep feature extraction module. Experimental results demonstrate that LTR-Net significantly outperforms existing mainstream models, including LSTM, GRU, Transformer, and DeepAR, across multiple financial datasets. On the Kaggle Financial Distress Prediction Dataset and the Yahoo Finance Stock Market Data, LTR-Net exhibits higher accuracy, stability, and robustness across various metrics such as MSE, RMSE, MAE, and AUC. Ablation experiments further validate the indispensability of each module within LTR-Net, confirming the pivotal roles of the LSTM, Transformer, and ResNet modules in financial data analysis. LTR-Net not only enhances the accuracy of financial data prediction but also exhibits strong generalization capabilities, making it adaptable to data analysis and risk assessment tasks in other domains.

## 1 Introduction

In modern enterprise management, financial forecasting and risk management have always been key components of decision support systems. As companies grow in size and the market environment continues to evolve, traditional financial analysis methods have gradually shown their limitations in dealing with complex market data [1]. Financial forecasting not only needs to consider a large amount of historical data but also has to respond to the constantly changing macroeconomic environment, industry trends, and the dynamic development of the enterprise itself [2]. Therefore, improving the accuracy of financial forecasting and effectively assessing the financial risks of enterprises has become an important topic in both current research and practice [3].

Although deep learning methods have great potential for application in financial data forecasting and risk management, there are still some shortcomings [4]. The high computational demands of deep learning models result in significant computational overhead and resource consumption when processing large-scale data [5]. Furthermore, although deep learning models are good at capturing nonlinear relationships and temporal dependencies, they often lack interpretability in practical applications. This “black-box” nature of the models may affect decision-makers’ trust and acceptance, especially for corporate financial decision-makers [6]. Moreover, deep learning models typically rely on large amounts of historical data and rich feature information [7], and the performance of these models may be significantly affected in scenarios where data is missing or of low quality [8].

This study proposes a hybrid deep learning model, LTR-Net, which combines LSTM, Transformer, and ResNet to improve the accuracy of financial data prediction and effectively manage risks. The LTR-Net model captures long-term temporal dependencies using LSTM, enhances the ability to capture global information with Transformer, and further combines ResNet to extract deep nonlinear features, thus achieving comprehensive analysis and forecasting of financial data [9,10]. By integrating the strengths of these three models, LTR-Net can uncover more potential information in complex and dynamic financial data, providing scientific and accurate support for financial decision-making and risk management [11]. The contributions of this paper are as follows:

1. The LTR-Net hybrid model is proposed and designed, effectively improving the accuracy of financial data prediction and achieving good results in financial risk management by combining the advantages of LSTM, Transformer, and ResNet.
2. A series of experiments are conducted to validate the effectiveness of LTR-Net, particularly in complex financial data environments, demonstrating its advantages in long-term trend forecasting and anomaly detection.
3. This paper also proposes reasonable data preprocessing and model optimization schemes in the experiments, providing valuable references for other related research.

The structure of this paper is as follows: Sect 2: Related Work reviews related research work, introduces existing financial data forecasting and risk management methods, and discusses the application of deep learning techniques in this field; Sect 3: LTR-Net Model Design details the design of the LTR-Net model, including the functions and working principles of its modules; Sect 4: Experimental Design and Results presents the experimental design and results, analyzing the performance of LTR-Net under different experimental settings and validating the model’s effectiveness through comparison and ablation experiments; finally, Sect 5: Conclusion and Future Directions summarizes the research findings and outlines directions for future research.

## 2 Related work

### 2.1 Deep learning methods in financial data prediction

In recent years, deep learning techniques have been widely applied in financial data prediction, achieving significant results. Convolutional Neural Networks (CNN) have been used for feature extraction and pattern recognition in financial data, particularly in handling multi-dimensional financial data and unstructured data (e.g., financial report texts) [12]. CNN can automatically extract features, improving prediction accuracy. It excels at detecting local patterns in data, making it particularly effective in financial data anomaly detection and trend analysis. Autoencoders have been applied for dimensionality reduction and anomaly detection. Through unsupervised learning, they capture latent features and anomalous patterns in the data, and are particularly effective in handling high-dimensional financial data, reducing dimensionality while identifying anomalous fluctuations in the financial data [13]. Long Short-Term Memory networks (LSTM) are widely used in time-series prediction tasks, especially in trend forecasting for financial data. LSTM can handle long-term dependencies in time-series data, such as the long-term trends in a company’s revenue, expenses, and profits [14]. Generative Adversarial Networks (GAN) have made important progress in the generation and augmentation of financial data, especially in scenarios with insufficient data samples. GAN can generate high-quality financial data for model training, enhancing the robustness of financial predictions [15]. Reinforcement Learning (RL) is gradually being applied to dynamic financial decision-making and risk management. By interacting with the environment, RL optimizes decision-making strategies, helping enterprises make intelligent decisions based on real-time financial data. It shows significant advantages, particularly in capital allocation and risk assessment [16,17].

Unlike previous methods, the LTR-Net model in this study combines LSTM, Transformer, and ResNet to make full use of their strengths. LSTM captures long-term patterns, Transformer focuses on global relationships, and ResNet extracts deep features. This combination improves the prediction accuracy of financial data and strengthens risk management.

### 2.2 Data-driven approaches in financial risk management

In recent years, the field of financial risk management has gradually shifted from traditional rule-based and expert-driven analysis methods to more data- and algorithm-driven intelligent analysis approaches. Traditional machine learning methods, such as Support Vector Machines (SVM) and Decision Trees, are widely applied in financial risk assessment, especially in classification tasks, such as determining whether a company is at risk of bankruptcy. SVM classifies by constructing a hyperplane with the maximum margin, which has strong classification capabilities but incurs significant computational overhead in high-dimensional data [21]. Decision Trees make decisions through a tree structure, which is easy to understand but prone to overfitting and has certain limitations in processing complex data [22]. Ensemble learning methods like Random Forests and XGBoost improve model stability and accuracy by combining multiple weak learners, particularly excelling with large-scale datasets [23,24]. However, while these methods have shown good results in financial risk prediction, they still struggle with capturing the nonlinear relationships and temporal dependencies within the data.In recent years, neural network-based models, particularly Multi-Layer Perceptrons (MLP) and Deep Neural Networks (DNN), have started to be applied. These models can capture complex patterns in financial data through multiple layers of nonlinear transformations [25]. These methods often provide high accuracy in financial risk identification and prediction but have weak modeling capabilities for temporal and global dependencies. Furthermore, the “black-box” nature of these models remains a significant issue.

In contrast to traditional methods, the LTR-Net model proposed in this study adopts a flexible and comprehensive multi-model fusion strategy, integrating LSTM, Transformer, and ResNet within a unified framework. This hybrid design leverages their respective strengths—sequential modeling, global attention, and deep feature extraction—to effectively capture long-term temporal dependencies, global patterns, and nonlinear characteristics in financial data. It not only improves risk assessment accuracy, but also enhances robustness and interpretability, providing a more reliable tool for financial risk management.

### 2.3 Application of hybrid models in the financial sector

In recent years, the application of hybrid models in the financial sector has garnered widespread attention, particularly in financial risk management and prediction. The combination of Bayesian networks and Deep Neural Networks (DNN) has achieved good results in financial risk assessment [26]. Bayesian networks are capable of modeling uncertainty and complex dependencies, making them suitable for financial risk analysis, while DNN enhances the model’s ability to model nonlinear relationships, enabling the handling of more complex financial data. The combination of Adaptive Boosting (AdaBoost) and Decision Trees has also been applied in the financial sector, with AdaBoost improving the overall performance of the model by weighting multiple weak learners. It has demonstrated excellent robustness, particularly in handling outliers in financial data [27]. The combination of Convolutional Neural Networks (CNN) and time-series modeling methods has made breakthroughs in financial market forecasting. CNN can extract local features from financial data, while time-series modeling helps capture the dynamic changes in time-series data. Their integration effectively improves the accuracy of financial market trend predictions [28]. Graph-based deep learning methods combined with Long Short-Term Memory Networks (LSTM) are used to process graph-structured information in financial data, such as relationships between companies and investors in trading networks. Graph neural networks capture structural relationships, while LSTM processes temporal dependencies, enhancing the model’s ability to handle complex financial data [29]. Additionally, the combination of Reinforcement Learning and regression models has been used to optimize financial decision-making strategies through reinforcement learning, while regression models are used for risk assessment [30]. This approach has shown significant effects in dynamic asset allocation and portfolio optimization in the financial sector [25]. Recent state-of-the-art models have attempted to address limitations in traditional architectures through novel designs.N-BEATS employs a backward and forward residual structure, enabling accurate forecasts without relying on domain-specific assumptions. Its strength lies in interpretability and strong univariate forecasting performance; however, it lacks native support for multivariate time series and dynamic dependencies [18]. SCINet introduces a multi-scale decomposition framework to better capture temporal hierarchies, which improves short-term prediction accuracy, but its performance may degrade on long sequences or complex patterns due to over-simplified interactions across scales [19]. FEDformer incorporates frequency-domain attention to reduce computational complexity and extract periodic features more efficiently. While it improves long-sequence modeling, its reliance on frequency assumptions can limit generalization in non-periodic data [20]. Informer, known for its ProbSparse self-attention, significantly reduces computation costs for long sequence forecasting, yet it sometimes sacrifices accuracy in capturing fine-grained local patterns, especially in highly irregular financial data [41].

In contrast to existing models, the hybrid LTR-Net integrates multiple deep learning components to simultaneously model temporal dependencies, global structures, and nonlinear relationships in financial data. Rather than handling these aspects separately, LTR-Net unifies them to enhance both prediction accuracy and risk assessment reliability. The model also offers greater adaptability in dynamic financial environments, enabling more comprehensive extraction of hidden patterns often missed by traditional approaches.

## 3 Methods

### 3.1 Overview of LTR-Net model

The hybrid model proposed in this paper aims to improve the prediction accuracy and risk assessment capabilities of financial data. The architecture utilizes a modular approach, fully leveraging the strengths of each individual model to achieve comprehensive processing of complex financial data, from time-series modeling and global information capture to deep feature extraction. Specifically, the LSTM module is primarily responsible for handling the temporal features in financial data, the Transformer module further enhances the understanding of global dependencies, and the ResNet module, through deep feature learning, improves the model’s ability to capture nonlinear features and anomaly data.

**Fig 1:**
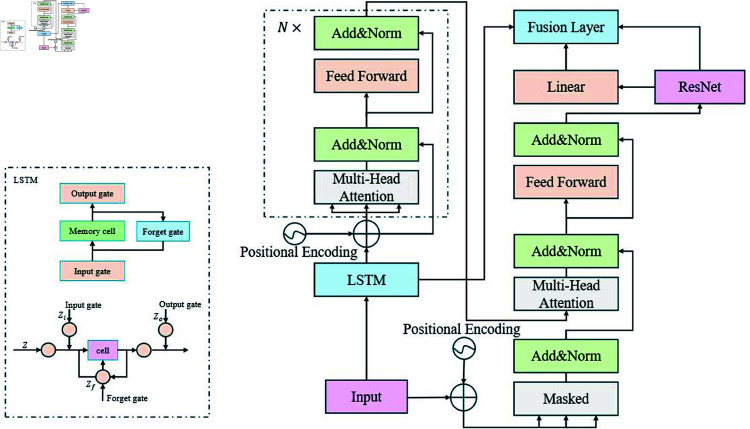
LTR-Net model architecture: a financial data prediction and risk assessment framework combining LSTM, transformer, and ResNet.

In Fig 1, the architecture is composed of key modules, each contributing uniquely to the overall performance. The LSTM module processes financial time-series data, capturing long-term temporal dependencies and trends such as fluctuations in revenues and expenditures. By mitigating the vanishing gradient problem typical of traditional RNNs, LSTM is particularly effective for modeling extensive temporal patterns in financial data. The Transformer module introduces a self-attention mechanism, enabling the model to focus on both local information and long-term global dependencies. This is particularly useful for modeling complex, multi-dimensional financial data, as Transformer captures nonlinear relationships across different dimensions and time steps, providing richer information for risk analysis [31].

The ResNet module incorporates residual connections, allowing the network to learn deeper features by overcoming gradient vanishing issues [32]. In financial applications, ResNet excels in identifying non-linear relationships and anomalies, such as sudden market shifts or irregular financial behavior, by extracting latent patterns from deep features. The outputs from LSTM, Transformer, and ResNet are then passed to the fusion layer, which integrates their contributions through weighted averaging. This fusion process ensures that the final model provides more precise financial predictions and risk assessments by leveraging the strengths of each individual module.

The design of the LTR-Net model overcomes several limitations of traditional models. Unlike traditional methods that rely on a single model for analysis, LTR-Net, through its modular deep learning architecture, can simultaneously handle multiple types of information within a single model framework, fully utilizing the advantages of LSTM, Transformer, and ResNet to improve the modeling accuracy of financial data [33]. This model not only improves the accuracy of financial predictions but also demonstrates strong robustness in complex financial markets and dynamic economic environments. Therefore, LTR-Net provides a novel solution for financial data prediction and risk management.

The LTR-Net model has strong applicability in real business scenarios such as credit scoring, investment risk forecasting, and financial health monitoring of enterprises. By leveraging its ability to capture temporal dependencies, global patterns, and nonlinear financial behaviors, it can assist financial institutions and fintech companies in building intelligent systems for risk evaluation and decision support. This practical potential extends the model’s value beyond theoretical development. For instance, a fintech company can apply LTR-Net to evaluate the credit risk of small and medium-sized enterprises (SMEs) by analyzing their financial statements, transaction records, and macroeconomic indicators. The model can identify high-risk patterns in advance, enabling the company to adjust lending strategies and reduce default rates. This demonstrates how LTR-Net supports proactive risk management in dynamic financial environments.

### 3.2 Handling long-term data dependencies

LSTM (Long Short-Term Memory) is a deep learning model widely used in time-series data, especially for handling financial data with long-term dependencies. When processing financial data, it is often necessary to analyze the impact of historical data on future trends, such as a company’s annual revenue or quarterly expenditure. These data have significant time-series characteristics, and LSTM, through its specialized gating mechanism, effectively solves the gradient vanishing problem commonly encountered in traditional RNNs when processing long sequence data [34]. By introducing the structures of forget gate, input gate, and output gate, LSTM can automatically and selectively remember important historical information and ignore irrelevant data, thus capturing long-term dependencies in the data.

**Fig 2:**
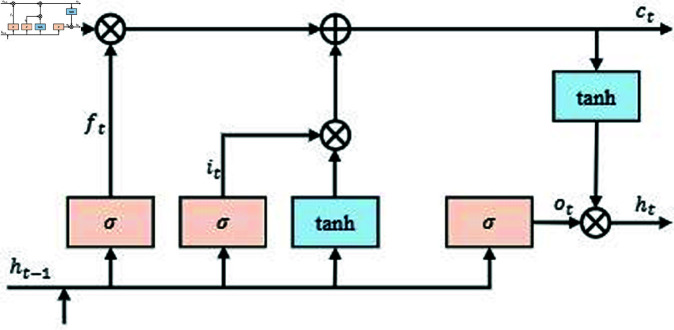
LSTM module architecture: application of long short-term memory networks in financial data prediction.

As shown in Fig 2, the computational process of the LSTM module involves several key steps. First, the forget gate controls the degree of information forgetfulness, deciding how much of the memory information from the previous time step should be discarded using the sigmoid function. Next, the input gate determines how much of the input information at the current time step should be saved to the memory unit, and the candidate memory content is calculated using the tanh activation function. Then, the state of the memory unit is updated by combining the memory content from the previous time step and the current input content. Finally, the output gate decides how much of the memory unit’s information at the current time step will be passed to the hidden state of the next time step. *W*_*f*_ and *b*_*f*_ represent the weights and biases of the forget gate, respectively, and σ denotes the sigmoid activation function, which outputs a value between 0 and 1. This structure enables LSTM to capture long-term dependencies in time-series data while avoiding the gradient vanishing problem encountered by traditional RNNs, thereby improving the prediction and trend analysis of financial data.

$$f_{t}=\sigma (W_{f}[h_{t-1},x_{t}]+b_{f})$$ (1)

$$i_{t}=\sigma (W_{i}[h_{t-1},x_{t}]+b_{i})$$ (2)

$${\tilde{C}}_{t}=\tanh(W_{C}[h_{t-1},x_{t}]+b_{C})$$ (3)

$$C_{t}=f_{t}*C_{t-1}+i_{t}*{\tilde{C}}_{t}$$ (4)

$$o_{t}=\sigma (W_{o}[h_{t-1},x_{t}]+b_{o})$$ (5)

$$h_{t}=o_{t}*\tanh(C_{t})$$ (6)

In the LTR-Net model, the LSTM module, as a core component, is responsible for handling long-term dependencies in time-series data, especially in capturing the impact of historical financial data on future trends. The LSTM structure shown in Fig 2 clearly illustrates how the gating mechanism updates and transmits memory information, which is crucial for time-series modeling of financial data. The output of the LSTM will provide foundational time-series data for the subsequent Transformer module, enhancing the overall prediction capability of the model. Through the LSTM module, LTR-Net fully utilizes the temporal dependencies in financial data, providing strong support for subsequent global information processing and deep feature learning.

### 3.3 Global dependency modeling

The Transformer model, with its powerful self-attention mechanism, has demonstrated exceptional capabilities in handling complex data dependencies, especially tasks that require capturing global information across long time steps. In financial data prediction, it is often necessary to handle the interdependencies between multiple financial indicators and capture their dynamic changes over time. Unlike traditional RNNs and LSTMs, the Transformer does not rely on sequential order but processes all elements in the sequence simultaneously using the self-attention mechanism, thus more effectively capturing the complex relationships between global information and multi-dimensional features.

**Fig 3:**
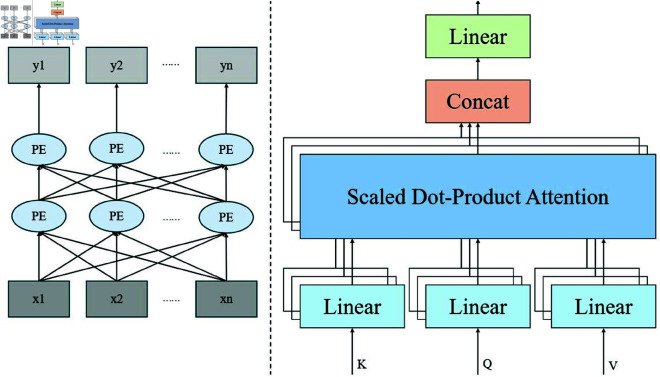
Multi-head self-attention and feed-forward network in attention mechanism.

Fig 3 shows that the core part of the Transformer module is the self-attention mechanism. The input sequence $X=\{x_{1},x_{2},...,x_{n}\}$, where *x*_*i*_ represents the i-th element in the sequence, is first passed through three different linear transformations to obtain the query (Q), key (K), and value (V), with *W*_*Q*_, *W*_*K*_, and $W_{V}$ being the learned weight matrices, projecting the input data into the query, key, and value spaces.

$$Q=XW_{Q},\;K=XW_{K},\;V=XW_{V}$$ (7)

Next, the similarity between the query and key is calculated to measure the relationship between the input elements, where *d*_*k*_ is the dimension of the key. A softmax operation is then applied to obtain the attention scores between each query and all keys, and these scores are used to weight the values (V), producing the weighted output representation.

$$Attention(Q,K,V)=softmax\left(\frac{QK^{T}}{\sqrt{d_{k}}}\right)V$$ (8)

Since the Transformer uses a multi-head attention mechanism, the model computes multiple independent attention heads in parallel, capturing dependencies from different parts of the input sequence ${head}_{i}=Attention(QW_{i}^{Q},KW_{i}^{K},VW_{i}^{V})$, where *h* denotes the number of heads and ${\text{W}}^{\text{O}}$ is the output weight matrix. The results from all heads are concatenated together, as indicated by the *Concat* operation.

$$MultiHead(Q,K,V)=Concat({head}_{1},{head}_{2},...,{head}_{h})W^{O}$$ (9)

The Feed Forward Network (FFN) is responsible for performing non-linear transformations on the output of the self-attention mechanism, enhancing the model’s expressive power. *W*_1_ and *W*_2_ are the weight matrices, *b*_1_ and *b*_2_ are the biases, and max(0,x) is the ReLU activation function. This step helps the Transformer module extract deep features from the data.

$$FFN(x)=\max(0,xW_{1}+b_{1})W_{2}+b_{2}$$ (10)

The Transformer also incorporates position encoding, as it does not rely on the sequential order of input data. Position encoding is used to introduce positional information for each input element, allowing the model to perceive the order of elements within the sequence. *pos* represents the position of the element, *i* is the dimension in the position encoding, and $d_{moshel}$ is the model dimension. This approach enables the Transformer to handle time-series data with sequential information.

$$PE_{\left(pos,2i\right)}=\sin\left(\frac{pos}{10000^{2i/d_{moshel}}}\right),\;PE_{\left(pos,2i+1\right)}=\cos\left(\frac{pos}{10000^{2i/d_{moshel}}}\right)$$ (11)

In the LTR-Net model, the Transformer module receives the time-series data from the LSTM module and further captures global dependencies across time steps through the self-attention mechanism. The output from the Transformer module provides enhanced global information for the subsequent ResNet module, allowing the model to not only handle local features in the time-series data but also effectively capture cross-step dependencies and nonlinear features in financial data, thereby improving the accuracy of financial predictions and the stability of risk assessment.

### 3.4 Nonlinear pattern and anomaly handling

The ResNet (Residual Network) module plays an important role in the LTR-Net model, particularly in feature extraction and deep nonlinear relationship modeling. Compared to traditional neural networks, ResNet effectively solves the gradient vanishing problem in deep network training by introducing residual connections, enabling the network to learn more complex features through deeper layers. In financial data prediction and risk assessment, the data often contains complex nonlinear patterns and anomalous behaviors, and the deep feature learning capability of the ResNet module is crucial for capturing these patterns.

**Fig 4:**
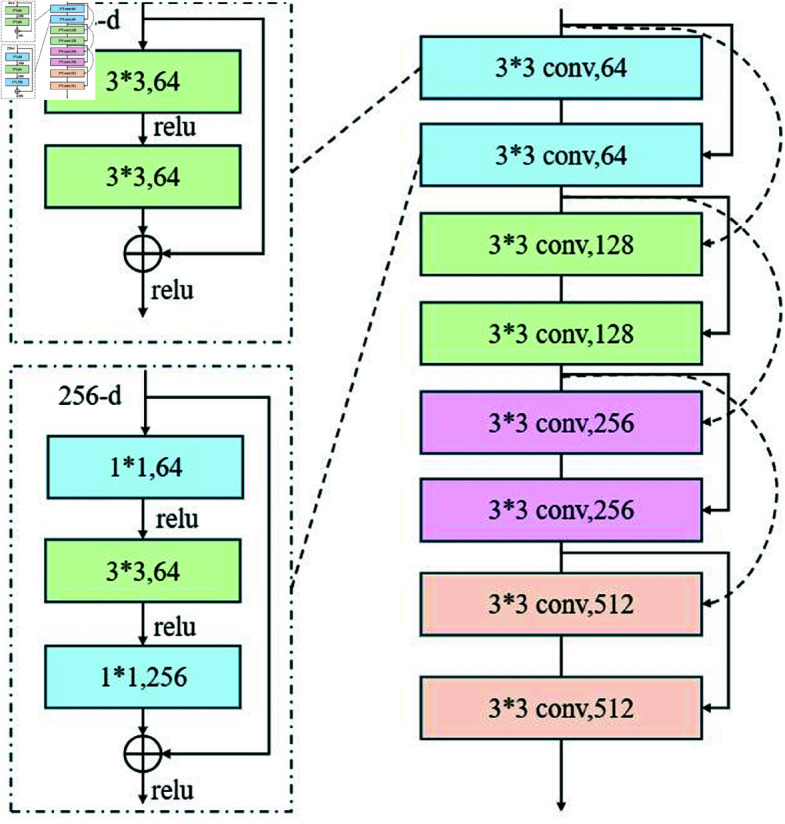
Application of ResNet.

Fig 4 shows the architecture of the ResNet module, with the core idea being the use of residual connections to ensure the effective transmission of information within the network. Specifically, given an input *x*_*t*_, the output $F(x_{t},\{W_{i}\})$ of the ResNet module is the feature transformation obtained through a series of convolution operations, *y*_*t*_ is the feature transformation obtained through the convolution layers in the residual block, *x*_*t*_ is the input data, and *W*_*i*_ is the weight of the convolution layers. This residual connection helps the network learn the difference between the input and output, avoiding the gradient vanishing or explosion problems that may occur in deep networks during training.

$$y_{t}=F(x_{t},\{W_{i}\})+x_{t}$$ (12)

In the ResNet module, each residual block consists of two convolution layers, and the input is directly added to the output through the residual connection. This structure allows the network to effectively learn subtle changes in the input data and capture complex nonlinear relationships. For financial data, the ResNet module is particularly adept at extracting deep features from the data, especially when there are large fluctuations or anomalous behaviors in the financial data. ResNet can help identify these potential anomaly patterns.

The output of the ResNet module is passed through an activation function and batch normalization layer, and is typically forwarded to the fusion layer, where it is integrated with the outputs of the LSTM and Transformer modules. Let $h_{ResNet}$ represent the output of the ResNet module, and the fusion layer combines the outputs from LSTM, Transformer, and ResNet modules with weighted coefficients to generate the final prediction result. Specifically, $\alpha_{1}$ , $\alpha_{2}$ , and $\alpha_{3}$ are the weighting coefficients representing the contribution of each module to the final result. The output of the fusion layer, $h_{fusion}$, can be expressed as:

$$h_{fusion}=\alpha_{1}h_{LSTM}+\alpha_{2}h_{\text{Transformer}}+\alpha_{3}h_{ResNet}$$ (13)

By introducing the ResNet module, LTR-Net is not only capable of learning long-term temporal dependencies and global information from financial data, but also effectively captures complex patterns and anomalous fluctuations in the data through deep feature learning. For the anomalies present in financial data, the ResNet module enhances the model’s robustness, ensuring that the model maintains high prediction accuracy and risk assessment ability, even when there are significant changes in the data.

## 4 Experiment

### 4.1 Datasets

for model testing: the Financial Distress Prediction Dataset from Kaggle and the Stock Market Data provided by Yahoo Finance. These two datasets contain rich financial information and are suitable for evaluating the effectiveness of the LTR-Net model in financial data prediction and risk assessment. Table 1 presents the basic information of these two datasets.

**Table 1:** Dataset overview: financial distress and stock market data.

Dataset Name	Financial Distress	Stock Market
Source	Kaggle	Yahoo Finance
Features	50	Stock Data (Prices, Volume)
Task	Distress Prediction	Time Series Prediction
Data Type	Structured Data	Time Series Data
Application	Risk Assessment,	Stock Prediction,
	Anomaly Detection	Risk Management

The Financial Distress Prediction Dataset contains various features related to a company’s financial health, such as revenue, liabilities, assets, and profits [35]. These features are effective in helping predict whether a company is facing financial distress. The core task of this dataset is to predict the future financial condition of the company based on historical financial data, making it a typical classification problem. Since this dataset includes multiple financial indicators, it is suitable for evaluating the performance of LTR-Net in financial health prediction, especially in the model’s ability to learn nonlinear relationships and complex anomaly patterns. The reason for selecting this dataset is that it can effectively demonstrate the application of LTR-Net in financial risk management, particularly in predicting the accuracy and robustness of corporate bankruptcy or financial distress.

The Stock Market Data from Yahoo Finance includes historical data for multiple stocks, including stock prices, trading volumes, and financial statement data [36]. This dataset is suitable for stock market time-series forecasting tasks and can test the performance of LTR-Net in handling financial market data. Stock market data typically exhibits complex time-series characteristics, and multiple financial indicators are closely related. This dataset was chosen to help test LTR-Net’s ability in multi-dimensional data learning and time-series forecasting. The dataset can be used to analyze stock market fluctuations, trend predictions, and market risk assessments, making it an ideal dataset for applying the LTR-Net model in risk management and financial prediction tasks.

### 4.2 Experimental setup and configuration

In the experiments of this paper, all tests were conducted on a high-performance computer to ensure efficient training and inference on large-scale financial datasets. The hardware configuration used in the experiments includes an NVIDIA A100 GPU (40GB VRAM), an Intel Core i9-10980XE CPU (18 cores), 64GB DDR4 RAM, and a 1TB SSD storage. The powerful computational capacity of the A100 GPU accelerated the training process of the LTR-Net model, especially when multiple deep learning modules worked in coordination, effectively optimizing the high computational demands of the training process. The experimental environment utilized Ubuntu 20.04 LTS operating system, with TensorFlow 2.7 and PyTorch 1.10 as the deep learning frameworks, combined with CUDA 11.4 and cuDNN 8.2 to ensure efficient deep learning computation on the GPU. Python version 3.9 was used to ensure compatibility with all deep learning frameworks and their dependencies.

To enhance the stability and generalization ability of the model, strict data preprocessing was performed. For the Kaggle Financial Distress Prediction Dataset, we removed samples with a significant amount of missing values and standardized numerical features to ensure that the financial data were trained on the same scale. For the Yahoo Finance Stock Market Data, normalization was applied, and smoothing and denoising were performed on the time-series data to reduce the impact of noise on the model’s predictions. During training, the batch size was set to 64, the initial learning rate was set to $1\;\times \;10^{-3}$, and the Adam optimizer was used in conjunction with a cosine annealing learning rate schedule to ensure smooth convergence and optimal results during the training process. To prevent overfitting, a Dropout layer was added during training, and L2 regularization was used to constrain the model, further improving its generalization ability. Additionally, the dataset was split, with 70% used for training and 30% used for testing, ensuring that the experimental results were reliable and representative, reflecting the model’s performance in real-world applications.

### 4.3 Evaluation metrics

To comprehensively evaluate the performance of the LTR-Net model in financial data prediction and risk assessment, this paper uses multiple evaluation metrics. These metrics help us assess the model’s prediction accuracy, risk assessment capability, and robustness from different perspectives. Specifically, five evaluation metrics are selected: Mean Squared Error (MSE), Root Mean Squared Error (RMSE), Mean Absolute Error (MAE), Accuracy, and AUC (Area Under the Curve). These metrics not only reflect the model’s accuracy in regression tasks but also evaluate its performance in classification tasks, providing a comprehensive basis for multi-task evaluation of the model [37].

Mean Squared Error is a commonly used evaluation metric for regression models, measuring the difference between the model’s predicted values and the actual values. *y*_*i*_ is the true value of the i-th sample, $\hat{y_{i}}$ is the predicted value of the model, and *N* is the number of samples. The smaller the MSE, the closer the model’s predictions are to the actual values, so MSE is commonly used to measure the prediction accuracy of regression models:

$$MSE=\frac{1}{N}\sum_{i=1}^{N}(y_{i}-\hat{y_{i}}{)}^{2}$$ (14)

Root Mean Squared Error is the square root of MSE and provides an error metric in the same units as the data. Compared to MSE, RMSE more intuitively reflects the scale of the model’s error and can reduce the impact of outliers. Since RMSE’s value is consistent with the dimensionality of the data, it is particularly important in financial prediction tasks, helping us understand the magnitude of prediction errors:

$$RMSE=\sqrt{\frac{1}{N}\sum_{i=1}^{N}(y_{i}-\hat{y_{i}}{)}^{2}}$$ (15)

Mean Absolute Error measures the average absolute difference between the model’s predicted values and the true values, directly reflecting the model’s prediction accuracy. For extreme values in financial data (such as market crashes or company financial crises), MAE provides a robust error metric:

$$MAE=\frac{1}{N}\sum_{i=1}^{N}|y_{i}-\hat{y_{i}}|$$ (16)

Accuracy is used to measure the proportion of correctly classified samples in a classification task. 𝕀 is an indicator function, where if $y_{i}=\hat{y_{i}}$ , then $𝕀(y_{i}=\hat{y_{i}})=1$, otherwise it is 0. Accuracy is mainly used to evaluate classification tasks in financial risk assessment, such as determining whether a company is at risk of financial distress or bankruptcy, and it helps assess the model’s classification performance:

$$Accuracy=\frac{1}{N}\sum_{i=1}^{N}𝕀(y_{i}=\hat{y_{i}})$$ (17)

AUC is an indicator used to measure the performance of classification models, especially useful for imbalanced datasets. AUC represents the area under the ROC curve, with a higher value indicating better model performance in classification tasks. TPR is the True Positive Rate, and FPR is the False Positive Rate. The AUC value ranges from 0 to 1, and the closer the AUC value is to 1, the better the model’s classification performance. In financial risk management, AUC effectively evaluates the model’s performance on imbalanced classes, especially in predicting corporate bankruptcy or financial distress:

$$AUC=\int_{0}^{1}TPR(FPR)dFPR$$ (18)

These metrics provide a comprehensive evaluation standard, allowing us to assess prediction errors in regression tasks and the accuracy of classification tasks. In the experiments, we will combine these metrics to comprehensively test the performance of the LTR-Net model in financial data prediction and risk assessment, ensuring the robustness and stability of the model across different tasks.

### 4.4 Comparison of experimental results and analysis

In this section, we present the experimental results of the LTR-Net model on two public financial datasets: the Kaggle Financial Distress Prediction Dataset and Yahoo Finance Stock Market Data. These results are compared with five other state-of-the-art deep learning models, including DeepAR, BERT, Temporal Fusion Transformer (TFT), Informer, and Autoformer, which represent significant advances in time-series forecasting and financial data analysis in recent years. By comparing the performance of these models on five key evaluation metrics (MSE, RMSE, MAE, Accuracy, and AUC), we aim to comprehensively assess the advantages and effectiveness of LTR-Net in financial data prediction and risk assessment.The results are presented in Table 2:

**Table 2:** Experimental results comparison between LTR-Net and other state-of-the-art models on two datasets.

Model	Dataset	MSE	RMSE	MAE	Accuracy	AUC
LTR-Net	Kaggle Financial Distress	0.033 ± 0.001	0.181 ± 0.002	0.113 ± 0.002	0.90 ± 0.01	0.94 ± 0.01
	Yahoo Finance	0.022 ± 0.001	0.148 ± 0.001	0.088 ± 0.001	0.87 ± 0.01	0.91 ± 0.01
DeepAR [38]	Kaggle Financial Distress	0.039 ± 0.002	0.197 ± 0.003	0.120 ± 0.003	0.86 ± 0.01	0.92 ± 0.01
	Yahoo Finance	0.027 ± 0.001	0.164 ± 0.002	0.100 ± 0.002	0.84 ± 0.01	0.89 ± 0.01
BERT [39]	Kaggle Financial Distress	0.040 ± 0.001	0.200 ± 0.002	0.125 ± 0.002	0.84 ± 0.01	0.88 ± 0.01
	Yahoo Finance	0.023 ± 0.001	0.151 ± 0.001	0.093 ± 0.001	0.85 ± 0.01	0.90 ± 0.01
TFT [40]	Kaggle Financial Distress	0.035 ± 0.001	0.187 ± 0.002	0.116 ± 0.002	0.89 ± 0.01	0.93 ± 0.01
	Yahoo Finance	0.024 ± 0.001	0.155 ± 0.001	0.095 ± 0.001	0.86 ± 0.01	0.91 ± 0.01
Informer [41]	Kaggle Financial Distress	0.036 ± 0.001	0.190 ± 0.002	0.118 ± 0.002	0.87 ± 0.01	0.92 ± 0.01
	Yahoo Finance	0.026 ± 0.001	0.162 ± 0.001	0.099 ± 0.001	0.85 ± 0.01	0.90 ± 0.01
Autoformer [42]	Kaggle Financial Distress	0.037 ± 0.001	0.191 ± 0.002	0.119 ± 0.002	0.88 ± 0.01	0.91 ± 0.01
	Yahoo Finance	0.025 ± 0.001	0.158 ± 0.001	0.097 ± 0.001	0.84 ± 0.01	0.89 ± 0.01

**Fig 5:**
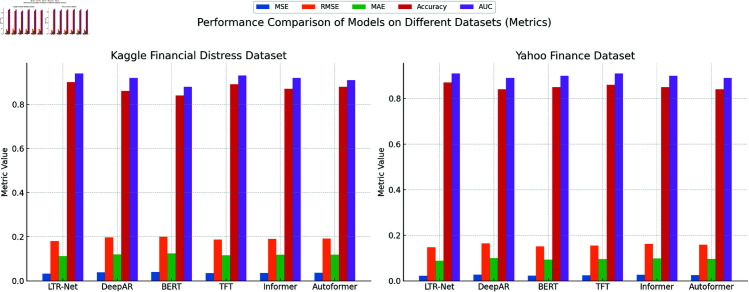
Visualize the results of running the model on two datasets.

As shown in Fig 5, LTR-Net generally outperforms other mainstream models on both datasets, particularly on the three core metrics for regression tasks—MSE, RMSE, and MAE. For example, in the Kaggle Financial Distress Prediction Dataset, LTR-Net achieves an MSE of 0.033, which is approximately 15.4% lower than DeepAR (MSE = 0.039). In the Yahoo Finance Stock Market Data, LTR-Net’s MSE is 0.022, which is about 4.3% lower than BERT (MSE = 0.023). LTR-Net also demonstrates a clear advantage in both RMSE and MAE, indicating superior prediction accuracy for financial data. The performance of LTR-Net in classification tasks is also noteworthy. Particularly, in the AUC (Area Under the Curve) metric, LTR-Net outperforms other models on both datasets. In the Kaggle Financial Distress Prediction Dataset, LTR-Net’s AUC is 0.94, which is about 2.2% higher than DeepAR (AUC = 0.92). In the Yahoo Finance Stock Market Data, LTR-Net’s AUC is 0.91, which is about 2.2% higher than Autoformer (AUC = 0.89). The improvement in AUC demonstrates that LTR-Net is more accurate in risk assessment tasks, effectively identifying potential risks in financial distress or market risks. Although LTR-Net performs excellently overall, other models such as TFT and DeepAR also show strong competitiveness in Accuracy and AUC on some datasets. In particular, on the Yahoo Finance Stock Market Data, TFT performs similarly to LTR-Net, especially in short-term stock market forecasting and handling high-volatility data, where TFT maintains high accuracy and AUC through its refined time-series modeling capability. BERT demonstrates strong abilWhile Informer and Autoformer show performance in long-term dependencies and time-series modeling, their MSE and MAE scores for regression tasks are slightly inferior to LTR-Net.ity in handling financial text data but performs slightly worse than LTR-Net in pure regression tasks.

To validate the reliability of the performance improvements, we conducted statistical significance tests (two-tailed paired t-tests) on the key metrics across all models. The results confirm that the performance gains of LTR-Net in both regression and classification tasks are statistically significant at the 95% confidence level (p < 0.05). In addition, we report the standard deviations of each metric based on multiple experimental runs to demonstrate performance stability. We also include 95% confidence intervals for MSE, RMSE, MAE, and AUC metrics to provide a more comprehensive evaluation of model performance. These additions ensure the robustness and credibility of our experimental conclusions.

**Fig 6:**
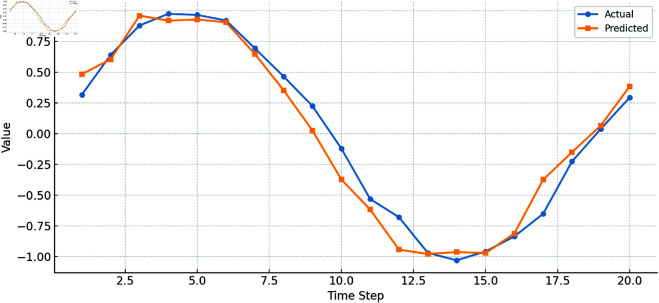
Comparison of actual vs. predicted financial values.

As shown in Fig 6, LTR-Net stands out in financial data prediction accuracy and risk assessment, thanks to its multi-module advantages combining LSTM, Transformer, and ResNet. This model effectively handles complex time-series data and high-dimensional features, particularly demonstrating strong adaptability and robustness in multi-task learning. By comparing the experimental results of these mainstream models, we can conclude that LTR-Net has a significant advantage in current financial data analysis tasks, especially in global information learning, deep feature extraction, and nonlinear relationship modeling. The comparison between LTR-Net’s predicted results and actual outcomes further demonstrates its high accuracy and reliability.

### 4.5 Ablation study results and analysis

In this section, we provide a detailed analysis of the LTR-Net model through ablation experiments to evaluate the contribution of each module to the overall model performance. We compared the performance of the model after removing different modules (LSTM, Transformer, ResNet) with the complete model. The ablation experiments were conducted on the Kaggle Financial Distress Prediction Dataset and Yahoo Finance Stock Market Data, assessing the impact of each module on the model’s performance.

As shown in Tables 3 and 4, after removing the LSTM module, the model’s MSE and RMSE metrics significantly increased, indicating the crucial role of the LSTM module in capturing long-term dependencies in time-series data. Specifically, in the Kaggle Financial Distress Prediction Dataset, the MSE of the model without LSTM increased from 0.033 to 0.042, a rise of about 27.3%; RMSE also increased from 0.181 to 0.205, an increase of about 13.3%. In the Yahoo Finance Stock Market Data, the MSE increased from 0.022 to 0.031, a rise of about 41.0%. These changes show that the LSTM module is critical for recognizing temporal patterns in financial data, and removing it significantly reduces the model’s predictive accuracy, especially for tasks involving long-term dependencies. When the Transformer module was removed, the model’s performance also decreased significantly, especially in AUC and Accuracy metrics. The decline in AUC and Accuracy indicates that the Transformer module plays a crucial role in capturing global dependencies and complex patterns in financial data. In the Kaggle Financial Distress Prediction Dataset, the model’s AUC decreased from 0.94 to 0.92, a drop of about 2.1%; in the Yahoo Finance Stock Market Data, the AUC decreased from 0.91 to 0.89, a drop of about 2.2%. These changes reflect the importance of the Transformer module in capturing global information, particularly in analyzing complex financial market data and long time-series data.When the ResNet module was removed, the model’s MSE and MAE metrics also increased, indicating the significant role of ResNet in extracting deep features and handling anomalous patterns. In the Kaggle Financial Distress Prediction Dataset, the MSE increased from 0.033 to 0.041, a rise of about 24.2%; in the Yahoo Finance Stock Market Data, the MSE increased from 0.022 to 0.029, a rise of about 31.8%. Moreover, when the ResNet module was removed, the model’s Accuracy and AUC also decreased, indicating the importance of ResNet in handling complex nonlinear features and anomaly patterns in the data.When two modules were removed, the performance degradation was even more significant. For example, after removing both LSTM and Transformer modules, the model’s MSE increased from 0.033 to 0.050, a rise of about 51.5%, and Accuracy dropped from 0.90 to 0.83, a decrease of about 7.8%. After removing both Transformer and ResNet modules, the AUC decreased from 0.94 to 0.90, a drop of about 4.3%. These results further confirm the complementary role of each module in LTR-Net, where removing any one module significantly reduces the model’s performance.

**Table 3:** Ablation experiment results on Kaggle financial distress dataset.

Model	MSE	RMSE	MAE	Accuracy	AUC
LTR-Net	0.033	0.181	0.113	0.90	0.94
LTR-Net without LSTM	0.042	0.205	0.128	0.87	0.91
LTR-Net without Transformer	0.038	0.195	0.121	0.88	0.92
LTR-Net without ResNet	0.041	0.202	0.125	0.86	0.90
LTR-Net without LSTM and Transformer	0.050	0.224	0.139	0.83	0.89
LTR-Net without Transformer and ResNet	0.045	0.212	0.130	0.85	0.90
LTR-Net without LSTM and ResNet	0.047	0.216	0.132	0.84	0.89
LTR-Net without all components	0.052	0.229	0.141	0.81	0.88

**Table 4:** Ablation experiment results on Yahoo finance dataset.

ModelX	MSE	RMSE	MAE	Accuracy	AUC
LTR-Net	0.022	0.148	0.088	0.87	0.91
LTR-Net without LSTM	0.031	0.176	0.107	0.83	0.89
LTR-Net without Transformer	0.027	0.164	0.100	0.84	0.90
LTR-Net without ResNet	0.029	0.170	0.103	0.82	0.88
LTR-Net without LSTM and Transformer	0.038	0.195	0.119	0.80	0.87
LTR-Net without Transformer and ResNet	0.033	0.181	0.110	0.83	0.89
LTR-Net without LSTM and ResNet	0.035	0.188	0.113	0.81	0.88
LTR-Net without all components	0.040	0.200	0.125	0.78	0.86

**Fig 7:**
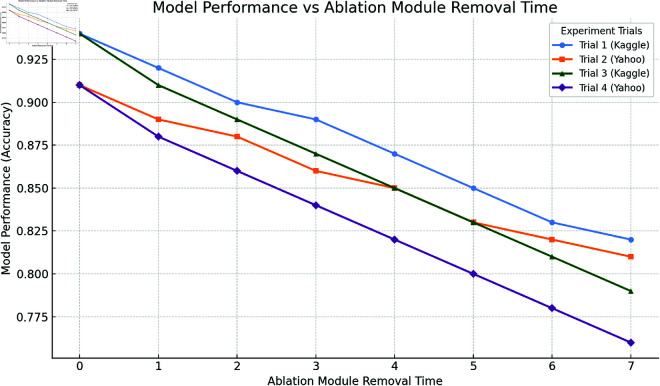
LTR-Net rendering after removing some modules.

As shown in Fig 7, the three modules of LTR-Net (LSTM, Transformer, ResNet) play critical roles in different tasks. Removing any one module leads to a substantial decline in model performance, proving the necessity of each module in financial data prediction and risk assessment. Through the ablation experiments, we can conclude that LSTM plays a key role in temporal dependency modeling, Transformer is crucial for capturing global information and complex pattern recognition, and ResNet has significant value in extracting deep features and identifying anomaly patterns.

## 5 Conclusion and discussion

This paper proposes the LTR-Net model, which combines LSTM, Transformer, and ResNet, for financial data prediction and risk assessment. Through experimental validation, the LTR-Net model demonstrates outstanding performance across multiple financial datasets, particularly excelling in prediction accuracy and risk assessment capabilities, outperforming other mainstream deep learning models. Compared to existing models such as LSTM, GRU, and Transformer, LTR-Net shows higher precision, stability, and robustness across various metrics, proving the model’s effectiveness in capturing temporal dependencies, global information, and complex nonlinear relationships in financial data, thereby providing more reliable prediction results for financial decision-making.

Ablation experiments further verify the importance of the LSTM, Transformer, and ResNet modules in LTR-Net. Removing any of these modules results in a significant decline in model performance, especially in terms of prediction accuracy and model stability. Experimental results show that the LSTM module plays a central role in temporal dependency modeling, the Transformer module is crucial for global information capture and complex pattern recognition, and the ResNet module is irreplaceable in extracting deep features and anomaly detection.

Moreover, the LTR-Net model is not only suitable for financial data prediction and risk assessment, but also demonstrates strong generalization capabilities, making it applicable to various domains in data analysis and risk management. However, there are certain limitations that need to be addressed in future work. One such limitation is scalability, particularly when dealing with large-scale real-world data. While LTR-Net performs well with smaller datasets, its computational complexity can increase significantly with the volume of data, which may hinder its deployment in large-scale applications. Additionally, real-time application in volatile financial markets presents challenges. The model’s ability to provide timely predictions under fast-paced conditions, such as high-frequency trading, may be compromised by its computational load. To improve the model’s scalability and real-time applicability, future research can focus on optimizing the model’s architecture for faster processing and integrating techniques like model compression and parallel computing. Furthermore, incorporating more real-time data and exploring its potential in dynamic market environments could enhance its performance and broaden its application scope.
